# Reply to Mr. Bampfield's Observations on the Use of Colchicum, Published in This Journal for November

**Published:** 1821-12

**Authors:** C. Scudamore


*Reply to* Mr. Bampfield's *Observations on the Use of Colchicum, published in this Journal for November.* By C. Scudamore, M.D.

THE observations contained in Mr. Bampfield's paper are so full of mis-statements, that, as far as they are personal, I should have felt it due to myself to pass them over in silence, if the writer had not, in a specious manner, supported his reflections with an apparent quotation from my treatise, and with studied calculations, so as to give to his opinions all the character of mathematical demonstration. It is, therefore, respecting the doctrines of which I have been an advocate, that I think it necessary to take up my pen on this occasion.

Mr. Bampfield begins with stating, that gout can be cured by smaller doses of colchicum than are exhibited by those who not only reprobate the use of what they prescribe, but deny it any "specific virtue." I shall avoid commenting upon all the parts of this singular paper, and carefully confine myself to the most particular passages in which I am concerned. He adopts as a quotation from my book the following words:—"The effects of colchicum in gout are altogether unsatisfactory." Is it just that an author shall be tried for his opinions by a garbled half passage,—by a few insulated words, to which his commentator annexes his own words and sentences, entirely straying away from the author's meaning, and the fair interpretation of his sentiments? It would surely have been the most regular proceeding to search for my sentiments on the subject in question in the last (the third) edition of my Treatise on Gout, &c. published in August 1819. The pretended quotation of these words is from page 193 of my second edition. To show the false stamp of the quotation, and the use made of it, I shall presently extract from this page the whole of the passage which bears upon the point in debate.

In the preceding pages, I had expatiated in praise of the autum. colchici, used in combination with purgative medicines. Speaking here, however, of colchicum in the state of tincture (digested in proof spirit,) and powder, I remark, "It was taken in free doses, but with effects altogether unsatisfactory, when used in either of these forms, and relied upon as the only medicine. I observed that the stomach was irritated; an increased fur of the tongue, and thirst, were produced; and no certain action of the bowels occurred." In the succeeding page, I remark on this passage, as follows: "Such was the statement which I offered in the former edition of this work, which I have quoted in order that I may lay before the reader, more clearly, my sentiments respecting this medicine. I am the more anxious to state the results of my increased experience with colchicum, because, thinking very highly of its powers in gout, under the mode of administration which I have detailed, I fear that I have not given to it the full credit which I think it deserves, in the preceding observations. I have not changed my general view of the nature of the remedy, as I shall proceed to show; but I think that a more particular explanation of the grounds of my praise and dispraise of this medicine than I have yet given, is due to the subject.

I cannot be so completely at variance with myself as to protest against the merits of colchicum, and to recommend it, in the same voice, in the manner that I am accused of doing. I may surely be allowed to prefer one preparation of colchicum to other preparations, and to employ the medicine upon my own principles, which differ from those which are acted upon by the practitioner who not only uses the more active forms of colchicum, but administers the medicine with a view to obtain its immediate, and more of its specific, action. In my desire to recommend to the profession the mildest preparation of colchicum with which I am acquainted, and that the treatment of gout, both in the fit and afterwards, should be conducted on extended general principles, I may possibly, in my first edition, have failed to express myself with sufficient clearness, as to the distinction which I wish to draw between colchicum and particular forms of colchicum, and between the dismissal of the present symptoms of the fit and the cure of the fit. If the free use of colchicum, *per se*, induce, in a remarkable manner, a much greater frequency in the return of the paroxysm, how can it be entitled to the praise of a specific cure for the gout? But, as I have fully detailed my view of the subject in my Treatise, I will forbear in this place from further argument in defence of my *principia*.

Mr. Bampfield's *Rule of Three* calls, however, for some exposition. His object is to show that I direct, in my prescriptions, much larger doses of colchicum than he employs in his successful treatment of a fit of the gout; and prefaces his calculations by stating that the London Pharmacopœia orders one ounce of the bulb to eight ounces of vinegar and one of spirit: but I find the proportions to be one ounce of the recent root to sixteen ounces of vinegar and one of spirit. He next endeavours to make it appear, first, that I direct, as a *medium* quantity for the twenty-four hours, one ounce of acetum colchici; and then states that this quantity is equivalent to $58\frac{1}{3}$ of colchicum in substance. He reckons that, in my ordinary treatment, I prescribe four times more of colchicum to be taken in the space of twenty-four hours than he administers; and that my *maximum* quantity is one ounce and a half, or, by his estimate, eighty grains of colchicum. Now see how the fact stands. In speaking in general terms of the draught which I recommend, I mention the varying doses of ʒj. ad ʒij.; and that the dose fixed upon may be given, at first, once in four, six, or eight hours, according to the effects produced. In the formula of the draught (p. 173), I express one drachm as the dose. I have not, in any part of my book, hinted at the employment of an ounce and a half in the twenty-four hours; and, in point of fact, I have never given a larger quantity than six drachms. It is also to be observed, that the acetum colchici in this draught, being combined with purgative ingredients, soon passes away from the stomach. In the last edition of my Treatise, I have detailed some experiments, made with a view to show the comparative strength and *modus operandi* of different gout medicines. As well as using the several preparations in the fluid form, I employed them in the state of extract,* obtained by evaporation of the menstrua over a water-bath. By such process of evaporation, one ounce of autum. colchici yielded $2\frac{1}{8}$ grains of a residue of soft consistence, all which substance I consider to be an active material. The wine of colchicum, prepared according to the directions of Sir Everard Home, yielded, of a residue of similar consistence, $16\frac{1}{2}$ grains, deduction being made for the residue derived from the wine itself, as shown by a separate experiment. In a series of expe-

* At that time, not having made sufficiently extensive trials of the properties of this extract as a medicine, I avoided any mention of its use; but I am now enabled to speak of it in the most favourable terms.

riments made upon the dog, for the sake of illustration, and reported in the third edition, I have distinctly proved the much greater mildness of action possessed by the autum. colchici and its extract, than belongs to the tincture, and the wine, and their extracts. I have deduced further consequent arguments in favour of the autum. and the extract; used, however, let me again observe, in combination with other medicines, and in conjunction with a general and comprehensive plan of treatment.

I shall here conclude my reply to Mr. Bampfield, which has necessarily been tedious to the reader, from the task imposed upon me of detailing so much misrepresentation and erroneous statement.

I merely wish to add, that, having satisfied my mind with the present refutation, I shall studiously avoid all future discussion of a controversial nature.

*Wimpole-street; Nov. 20th,* 1821.

---

